# Evaluation of antimicrobial and non-steroidal anti-inflammatory treatments for BRD on health and welfare in fattening bulls: a cross-sectional study

**DOI:** 10.1080/01652176.2024.2347928

**Published:** 2024-05-06

**Authors:** Naod Thomas Masebo, Giovanna Marliani, Flavia Shannon Del Re, Laura Abram, Damiano Cavallini, Marco Di Pietro, Andrea Beltrame, Eliana Schiavon, Marilena Bolcato, Joaquin Hernandez Bermudez, Arcangelo Gentile, Joana G. P. Jacinto

**Affiliations:** a Department of Veterinary Medical Sciences, University of Bologna, Ozzano Emilia, Bologna, Italy; b School of Veterinary Medicine, Wolaita Soddo University, Wolaita Soddo, Ethiopia; c Virbac, Italy Milano;; d Bovine Practitioner, Verona, Italy; e Istituto Zooprofilattico Sperimentale delle Venezie, Legnaro, PD Legnaro, Italy; f Departamento de Patologia Animal, Universidade de Santiago de Compostela Campus Universitario, Lugo, Spain

**Keywords:** Bovine respiratory disease, beef, cattle, M*ycoplasma bovis*, NSAID, tulathromycin

## Abstract

Our study aimed to evaluate the effect of different treatments for BRD on health and welfare in fattening bulls. A total of 264 bulls were enrolled. Welfare was assessed on day 2 (T0) and day 15 (T1) after arrival. A decrease in the welfare level was observed from T0 to T1. All bulls were inspected clinically at T0 and T1 revealing an increase of skin lesions and lameness in T1. In both periods, a high incidence of respiratory disease was observed. A prevalence of 79.55% and 95.45% of *Mycoplasma bovis* using RT-PCR and culture at T0 and T1 respectively was observed. Blood samples were collected for haematology at T0 and T1. At T0, 36 animals were individually treated for BRD with an antimicrobial (IT), 54 received a metaphylactic treatment with tulathromycin (M), 150 received a metaphylactic treatment with tulathromycin plus a second antimicrobial (M + IT) whereas 24 were considered healthy and therefore not treated (NT). Additionally, 128 were treated with a non-steroid anti-inflammatory (NSAID). Neutrophils of M + IT were significantly higher than groups NT and M and the lymphocytes of M + IT were significantly lower than that of IT. White blood cells, neutrophils and N/L ratio of animals treated with an NSAID was significantly higher than that not treated. Lung inspection of 172 bulls at the abattoir indicated that 92.43% presented at least one lung lesion. A statistically significant effect of the NSAID treatment on the lung lesions was observed. Our findings indicate that BRD was a major welfare and health concern and evidence the difficulties of antimicrobial treatment of *M. bovis*.

## Introduction

Beef production systems in the European Union (EU) differ in feeding management, housing, and in age and weight at slaughtering. In Italy, intensive fattening beef cattle management consists of indoor housing where cattle are managed more efficiently and fed to gain more weight than in extensive production systems. The specialised fattening units tend to be located in the Po Valley region and the stocked cattle are often imported from France. This production system has some critical points, such as health status of the newly received cattle, risk of respiratory and digestive disorders, and management practices that may impair meat quality (EFSA Panel on Animal Health and Welfare (AHAW) 2012).

Bovine respiratory disease syndrome (BRD) is one of the most high-cost disease and risk factor for the development of poor welfare in beef cattle all over the world particularly considering intensive systems (Chai et al. 2022; Cortes et al. 2021; Smith 2022). It is responsible for increased mortality rates and costs of treatment, reduced feed efficiency, and lower carcase quality (Padalino et al. 2021; Pratelli et al. 2021; Compiani et al. 2014). BRD affects the lower respiratory tract (bronchopneumonia, pneumonia) or/and upper respiratory tract (rhinitis, tracheitis, bronchitis) (Pratelli et al. 2021). It is multi-factorial, with a variety of physical and physiological stressors (Peel 2020). Transportation, climate change, temperature difference and also a new farm environment play a significant role as predisposing factors for BRD by favouring pathogen transmission and stress-induced susceptibility (Padalino et al. 2021; Smith 2022). The complexity of the interactions and time between these predisposing factors make BRD management and control challenging. The most common pathogens associated with BRD in beef cattle are *Pasteurella multocida*, *Mannheimia haemolytica*, *Histophilus somni*, *Mycoplasma bovis*, bovine herpesvirus type 1 (BoHV-1), bovine adenovirus (BAdV), bovine viral diarrhoea virus (BVDV), bovine coronavirus (BCoV), bovine respiratory syncytial virus (BRSV), and bovine parainfluenza 3 virus (Jelinski et al. 2020; Cirone et al. 2019). In particular, *M. bovis* infections are associated with chronic pneumonia and polyarthritis syndrome, otitis media, conjunctivitis and meningitis (Prysliak et al. 2011). Indeed, *M. bovis* is a bacterium of the respiratory microbiota that can become pathogenic under subsequent stressful situations (Tortorelli et al. 2017). BRD management and control is usually based on the administration of antimicrobials and anti-inflammatory drugs as a metaphylactic treatment and/or for individual treatment of clinically affected animals (Pratelli et al. 2021; Compiani et al. 2020; Moore et al. 2014). However, high antimicrobial usage and the risk of antimicrobial resistance are global issues of great concern for both human and animal health. The necessity to reduce the use of antimicrobials in animal food production sectors is highlighted as they play a significant role in the rise of antimicrobial resistance (Santinello et al. 2022).

In the present cross-sectional observational study, we aimed to evaluate the effect of antimicrobial and anti-inflammatory treatments for BRD on health and welfare, on newly introduced beef cattle in a commercial fattening unit of Limousine bulls affected by high prevalence of BRD due to *M. bovis.*

## Materials and methods

### Ethical approval

This study did not require official or institutional ethical approval as it was not experimental, but rather part of the routine of clinical and pathological veterinary diagnostics and procedures in a commercial fattening unit. All animals in this study were examined with the consent of their owners and handled according to good ethical standards.

### Housing, management and animals

The observational study was performed in a commercial fattening unit of Limousine bulls imported from France located in the province of Modena (Po valley region, Italy) from November 2021 to May 2022. This farm had a history of BRD *M. bovis*-related in the last production cycles. The study was conducted in a barn housing 264 animals. The barn was semi-closed and well-ventilated with curtained sidewalls. The barn had 44 pens in a free stall system with a capacity of 6 animals per pen (Supplementary Figure S1; https://academic.oup.com/jas/article/91/11/5455/4731491?login=true). A pen had a dimension of 18.4 m^2^. Each animal had a space of 3.06 m^2^ and a feeding space of 45 cm. The feeders were placed on one side along the manger. The pens were built adjacent to each other and were separated by iron bars, allowing interaction of animals in adjacent pens. The flooring was slatted and underneath there was a pit for manure collection. Before placing the animals in their respective pens, it was cleaned with a pressure washer and disinfected.

A total of 264 animals arrived to the fattening unit in numerically heterogeneous groups weekly-based with a total of 6 groups over the course of 6 weeks. These animals originated from various farms across France, encompassing different regions within the country. The majority of these bulls were primarily raised either on pastures or in indoor free stall systems with straw bedding. Before their arrival in Italy, the bulls spent one day in a selection centre in France, where they were separated on the base of their health status, age, and body weight in order to create homogeneous groups of animals. At arrival to the fattening unit in Italy, the bulls were on average 11 months old and weighed an average of 400 kg. At arrival, all animals were vaccinated with live attenuated virus of bovine viral diarrhoea virus (Rispoval D-Bvd®, Zoetis, Italy) and live vaccine of bovine herpesvirus type 1 (Bovilis IBR Live marker®, MSD Animal Health, Italy) for infectious bovine rhinotracheitis and were treated subcutaneously with Ivermectin (Ivomec®, Boehringer Ingelheim Animal Health, Italy). A vaccine booster was given four weeks after the first administration. No quarantine period was performed.

At the arrival, animals were fed an adaptation diet in order to reduce dietary stressors (Supplementary Table S1). The total mixed ration (TMR) was fed ad libitum and fresh clean water was always available. The TMR diet was fresh sampled in different locations (beginning, middle and end of the feeding line) at day 2 (T0) and day 15 (T1) after the arrival of animals to the fattening unit. Analytical TMR analyses were performed at the University of Bologna feed analysis laboratory according to the methodology described in previous studies (Mammi et al. 2022).

The production cycle lasted 5 months. During this period, 14 bulls had to be euthanized due to severe respiratory disease that did not respond to therapy. The other 250 bulls finished the cycle and were slaughtered with around 600 kg.

### Welfare assessment

The welfare assessment was carried out at T0 and T1 using an adapted version of the Italian protocol for the assessment of beef cattle welfare included in the ClassyFarm system (Bertocchi et al. 2020) as previously described (Masebo et al. 2023). The protocol utilised in the study consisted of a comprehensive list of 34 items, which were categorised into three main sections: A-farm management and staff training, B-housing and equipment and C-animal-based indicators. Each item was evaluated using a 2- or 3-point scoring system, with scores of 1 indicating inadequacy, scores of 2 indicating acceptability, and scores of 3 indicating optimisation. To calculate the overall score for the welfare, the scores obtained for each item within the areas were summed. A contribution of 50% was assigned to areas A and B, while the remaining 50% was assigned to area C. The resulting scores were then converted into percentages. Specifically, scores below 59% were categorised as poor status (low), scores between 60% and 80% were categorised as medium status (medium), and scores above 80% were categorised as good status (high).

### Clinical examination

An inspective pen-based clinical examination was performed for all animals (*n* = 264) at T0 and T1. It consisted in a 10 min-long observation with the observer standing among the animal in the pen. The following parameters were assessed: mental status, body condition score (BCS), skin lesions, locomotion score, respiratory findings, nasal discharge, ocular discharge, faecal consistency, and other eventual abnormalities. Four different mental status were considered: alert, dullness, stupor, and coma (Lorenz et al. 2011). The BCS was performed based on the Guide to Body Condition Scoring Beef Cows and Bulls, Kansas State University (Farney et al. 2016) and the locomotion score based on the Zinpro Step-Up Beef Cattle Locomotion Scoring System. Respiratory clinical findings included respiratory pattern, respiratory frequency, nasal discharge, ocular discharge, cough, and ear position (Baumgartner and Wittek 2017). The type of nasal discharge was classified as following: absent or present; if present monolateral or bilateral; mucous, haemorrhagic or purulent. The type of ocular discharge was classified as following: absent or present; if present monolateral or bilateral; mucous or purulent.

All data were recorded using a schematic table per pen (Supplementary Table S2). An animal was considered to be affected by BRD if it had at least two abnormal findings associated with the respiratory system (i.e. cough and nasal discharge; abnormal type of breath and cough; abnormal type of breath and nasal discharge).

### Blood analysis

Blood samples from 88 animals were collected for haematological investigation at T0 and T1. Animals were blindly randomised by an operator on the basis of ear tag number prior to inspection. Two animals were chosen randomly from each pen at T0, and the same subjects were re-sampled at T1. The samples were transferred into vacuum tubes containing EDTA anticoagulant for a complete blood count (CBC) and then into citrate tube for fibrinogen analysis. The following parameters were analysed: erythrocyte (RBC), haemoglobin, haematocrit (HCT), mean corpuscular volume (MCV), mean corpuscular haemoglobin (MCH), mean corpuscular haemoglobin concentration (MCHC), red blood cell distribution width (RDW), platelets (PLT), leucocytes (WBC), neutrophils, monocytes, lymphocytes, eosinophil, basophils and fibrinogen.

### RT-PCR and culture for Mycoplasma bovis

Deep nasal swabs were performed using MWE Pharyngeal Dryswabs (Ref:MW128, MWE®, UK) for RT-PCR and culture for *M. bovis*.

Deep nasal swabs in a pool of three samples were obtained from all 264 animals at T0 (total of 88 pools) to perform a qualitative RT-PCR for the detection of *M. bovis*. To collect the nasal swabs, animals were contained and the nostrils cleaned with paper before performing swabbing to avoid contamination. The nasal swabs were stored in dry collection tubes and analysed within 12 h after sampling. DNA extraction was performed using the Maxwell 16 LEV Blood DNA kit and Maxwell 16 Instrument, following the instructions provided by the manufacturer (Promega). A qualitative RT-PCR for the detection of *M. bovis* was used. The extracted DNA was then subjected to amplification through a PCR targeting the 16S-rDNA region and analysed using denaturing gradient gel electrophoresis (DGGE), following a previously established protocol (McAuliffe et al. 2005). The identification of different *Mollicutes* genera and species was accomplished by directly comparing the lane of interest with the reference strain profiles. To investigate the presence of *M. bovis* DNA in the collected swabs, total DNA was extracted from a portion of the corresponding transport medium. This DNA was then amplified using a specific PCR protocol for *M. bovis* (Butler et al. 2001) and analysed through electrophoresis in a 1% agar gel.

Deep nasal swabs in a pool of two samples were obtained from 88 bulls at T1 (total of 44 pools) for *Mycoplasma* culture. The 88 animals were the same sampled also for haematological investigation. To collect the nasal swabs, animals were restrained and the nostrils were cleaned with paper towels before performing swabbing to avoid contamination. The nasal swabs were stored in dry collection tubes and then immersed into 2 mL of Mycoplasma liquid medium (ML; Mycoplasma Experience Ltd., Bletchingley, UK) and maintained at 4^0^C until arrival to the laboratory. *Mycoplasma* cultivation and isolation were then performed in ML and PPLO (Pleuro-Pneumonia like Organisms) broth media. Mycoplasma cultivation and isolation were then performed as previously described (Catania et al. 2020). Briefly, the inoculated cultures were incubated at 37 °C with 5% CO_2_ for at least 7 days. The broths were checked daily up to 14 days to detect any change in colour or turbidity. Broths that showed any change were immediately inoculated onto a plate of semisolid Mycoplasma agar medium (MS; Mycoplasma Experience). Alternatively, broths that did not show any change were plated onto agar medium at the end of the observation period. If no colonies grew after 14 days, the sample was considered negative.

As the present study was an observational study in a commercial setting, there were some economic limitations. It was therefore decided to focus on testing for *M. bovis*, the pathogen historically associated with BRD outbreaks in the fattening unit.

### BRD antimicrobial treatment

The timing and the type of treatment was decided based on the findings obtained by clinical examination, decision of the farm practitioner and *M. bovis* testing. Four different treatments were considered. M, consisting of a metaphylactic treatment with tulathromycin (Tulissin®, Virbac, Italy) at T0. M + IT, consisting of a metaphylactic treatment with tulathromycin at T0 with a second antimicrobial treatment within the first 15 days on farm. IT, consisting of targeted individual treatment with an antimicrobial other than tulathromycin. Finally, NT, consisting of no antimicrobial treatment. M treatment was initiated when the prevalence of BRD in the arrival groups exceeded 20%. M + IT was initiated if an animal developed severe clinical signs of BRD within the first 15 days on the farm, despite having previously received M treatment. IT was initiated after a clinical diagnosis of BRD. NT was used in healthy animals.

### BRD non-steroid anti-inflammatory drugs treatment

At T0 or in the immediately following days, bulls were treated with non-steroid anti-inflammatory drugs (NSAID) if deemed necessary by the clinical findings (i.e. severe respiratory distress/abnormal mental status).

### Lung inspection at abattoir

The fattening period was five months and 250 bulls were slaughtered with a body weight of approximately 600 kg. A lung examination was carried out on 172 bulls. A lung score based on an estimation of the extension of diseased parenchyma was applied as following: no evidence of parenchymal alteration (healthy); inflammatory lesions affecting 1 to 25% of the parenchyma (mild pneumonia); inflammatory lesions affecting 25% to 50% of the parenchyma (moderate pneumonia); inflammatory lesions affecting more than 50% of the parenchyma (severe pneumonia) (Supplementary Figure S2).

### Statistical analysis

Data were entered into a statistics program (JMP Pro 17). Descriptive statistics were generated mean ± standard deviation (S.D.) and/or standard error (S.E.), median and range for continuous data, and count and percentage for categorical data. For continuous variables, normality was tested by Shapiro-Wilk test and non-normally distributed variables were Box-Cox transformed before the analysis. The evaluation of differences between the use/type of antimicrobial and anti-inflammatory treatment was undertaken using the Mixed Model Procedure. Each animal was set as experimental unit within the anti-inflammatory use, or antimicrobial use, depending on the model tested, arrival group, and pen as nested factors. The use/type of antimicrobial (NT, IT, M, IT + M) or the anti-inflammatory use (Y/N) treatment was implemented as a fixed effect in separate models. The day 2 (T0) was set as a covariate in both models. After the analysis, normal distribution of the data was checked again for the resulting residuals. Means are reported as least square mean and pairwise multiple comparisons were performed using Tukey-test as a post hoc test when a significance was detected. The nominal logistic model was used for categorical variables using the same discriminant as before mentioned. A *p*-value ≤ 0.10 was considered a tendency; a *p*-value ≤ 0.05 was considered statistically significant; and a *p*-value ≤ 0.01 was considered highly significant.

## Results

### Welfare assessment

Table 1 shows the results of the welfare assessment at T0 and at T1. At T0 the total welfare was 79.04% (medium). Data obtained for Area A, B and C were 70.45% (medium), 65.17% (medium) and 90% (high) respectively. At T1, a decrease in total welfare was observed (76.47%; medium). Even though there was an increase in Area B (68.57%), a decrease in total welfare was noticed when compared to T1 due to a decrease of Area C score (80%).

**Table 1.** Descriptive statistics of the welfare assessment of 264 beef cattle from an Italian beef fattening unite.

Item	Assessment at T0	Classification at T0	Assessment at T1	Classification at T1
Total welfare	79.04%	Medium	74.73%	Medium
Area A (Farm management and staff training)	70.45%	Medium	70.45%	Medium
Area B (Housing and facilities)	65.17%	Medium	68.57%	Medium
Area C (Animal-based indicators)	90%	High	80%	Medium

### Clinical examination

At T0 the following clinical findings expressed on percentage of affected animals were recorded as following: 1.51% of skin lesions, 0.75% of lameness, 0.75% of diarrhoea, 34% of coughing, 48.86% of nasal discharge, and 6.81% of ocular discharge. At T1 an increase in animals with integument lesions was observed (44.69%). Most of these were alopecic lesions in the neck. In addition, a slight increase in lameness (1.15%) and a moderate increase of coughing (52.65%) was noticed. Contrarily, a decrease in diarrhoea (0%) and nasal discharge (41.28%) were observed. More details are presented in Table 2.

**Table 2.** Clinical findings of the 264 beef cattle.

Item	Assessment at T0	Assessment at T1
Integument lesions (%)	4(1.51%)	118(44.69%)
Lameness (%)	2(0.75%)	4(1.15%)
Diarrhea (%)	2(0.75%)	0(0%)
Coughing (%)	90(34%)	139(52.65%)
Nasal discharge (%)	129(48.86%)	109(41.28%)
Ocular discharge (%)	18(6.81%)	4(1.15%)

### RT-PCR and culture for Mycoplasma bovis

At T0 70 out of 88 pools (79.55% ± 4.3%) were tested positive at RT-PCR for *M. bovis*. At T1 42 out of 44 pools (95.45% ± 3.14%) resulted positive at the culture of *M. bovis.*

### BRD treatments

An antimicrobial treatment was started on 240 animals by the local veterinarian at T0 or in the immediately following days. The following antimicrobial treatments were performed: 54 bulls received M at T0, 150 received M + IT, 36 animals received IT and 24 received NT (Supplementary Table S3).

At T0 or in the immediately following days, 128 animals were treated with NSAID (Supplementary Table S4).

### Lung lesions at abattoir

**Figure 1.**
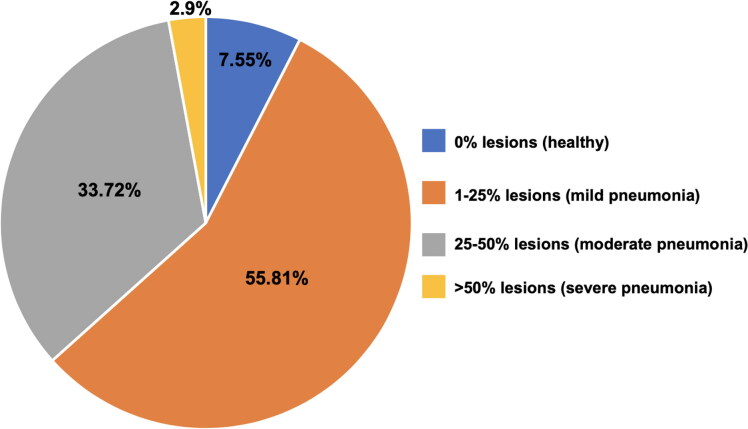
Lung lesions retrieved at the abattoir.

Figure 1 depicts the distribution of the lung lesions observed at abattoir. The most prevalent condition was mild pneumonia, observed in 96 animals (55.81% ± 3.79%). Moderate pneumonia was observed in 58 animals (33.72%). Severe pneumonia was observed in 5 animals. Only 13 animals (7.55%) could be considered completely normal.

### Effect of the different antimicrobial treatments on blood analysis, clinical findings and lung at abattoir

The effect of the different antimicrobial treatments at T0 on the blood analysis are presented in Table 3. At T1, there was a statistically significant effect (*p*-value ≤ 0.05) on the neutrophils, lymphocyte counts, and their ratio. The neutrophil count of the group M + IT was significantly higher than that of groups NT and M. Furthermore, the lymphocyte count of M + IT was significantly lower than that of IT. Consequently, the ratio N/L was significantly higher in M + IT compared to the other groups.

**Table 3.** Effect of the different antimicrobial protocols for BRD at T0 on the blood analysis at T1.

Blood parameters		Days								
		T0				T1				
		Treatment protocol				Treatment protocol				
		IT	M	M + IT	NT	IT	M	M + IT	NT	*p*
RBC (M/µL)	Mean ± SD	9.79 ± 1.44	9.32 ± 1.44	10.06 ± 1.26	8.66 ± 1.38	9.92 ± 1.15	9.79 ± 1.06	9.98 ± 1.09	9.18 ± 1.33	0.76
HGB (g/dL)	Mean ± SD	11.55 ± 0.9	12.08 ± 0.82	12.38 ± 1.26	10.72 ± 1.47	11.45 ± 0.81	12.2 ± 0.98	12.18 ± 1.17	11.33 ± 1.61	0.33
HCT (%)	Mean ± SD	38.93 ± 3.45	39.61 ± 3.66	40.13 ± 4.43	35.82 ± 5.68	38.46 ± 3.49	40 ± 3.73	39.45 ± 4.06	38.25 ± 4.9	0.68
MCV (fL)	Mean ± SD	40.15 ± 3.19	41.72 ± 3.15	40.17 ± 2.87	41.43 ± 3.18	38.91 ± 2.17	40.99 ± 2.76	39.64 ± 2.66	41.78 ± 1.75	0.09
MCH (pg)	Median [Min.–Max]	11.6 [10.9–13.15]	12.8 [11.85–13.5]	12.2 [11.8–12.9]	12.1 [11.8–12.9]	11.35 [10.8–12.78]	12.65 [11.63–13]	12.1 [11.6–12.75]	12.4 [11.73–12.95]	0.7
MCHC (g/dL)	Mean ± SD	29.72 ± 1.04	30.55 ± 1.14	30.91 ± 1.19	29.95 ± 1.12	29.88 ± 1.6	30.41 ± 1.45	30.9 ± 1.05	29.62 ± 1.09	0.19
RDW (%)	Mean ± SD	24.41 ± 1.61	23.62 ± 2.25	24.22 ± 1.69	23.65 ± 3.89	24.22 ± 1.45	24.12 ± 1.83	24.06 ± 1.55	23.45 ± 2.92	0.41
PLT (K/µL)	Median [Min.–Max]	338 [303–447.5]	289 [192.5–402.5]	227 [128–333]	265.5 [174.5–319.75]	347 [158.75–469.75]	406 [235–524]	296 [133–525.5]	141 [94.5–260]	0.29
WBC (K/µL)	Median [Min.–Max]	8.69 [7.59–11.09]	8.51 [7.07–9.395]	8.79 [7.35–10.28]	7.36 [7.08–8.41]	12.7 [9.1–14.52]	10.15 [8.14–11.69]	10.4 [8.52–12.97]	8.95 [6.82–12.23]	0.13
NEU (K/µL)	Median [Min.–Max]	3.32 [2.43–3.71]	2.94 [2.57–3.77]	3.65 [2.95–4.27]	2.35 [2.00–2.91]	4.27 [3.13–6.7]^ab^	3.21 [2.62–4.15]^b^	4.48 [2.93–6.77]^a^	3.32 [2.23–4.02]^b^	0.05
MONO (K/µL)	Median [Min.–Max]	1.3 [0.94–1.4]	1.04 [0.84–1.26]	1.29 [1.0275–1.5725]	1.15 [0.9325–1.3625]	1.18 [0.96–1.53]	1.07 [0.83–1.35]	1.12 [0.87–1.4]	1.16 [0.87–1.23]	0.81
LYM (K/µL)	Mean ± SD	4.23 ± 0.97	3.94 ± 0.96	3.71 ± 1.43	3.91 ± 0.8	5.87 ± 1.75^a^	4.68 ± 1.82^ab^	4.05 ± 1.7^b^	4.58 ± 1.37^ab^	0.04
EOS (K/µL)	Median [Min.–Max]	0.13 [0.03–0.33]	0.05 [0.04–0.14]	0.07 [0.02–0.19]	0.12 [0.08–0.23]	0.23 [0.08–0.44]	0.22 [0.09–0.4]	0.23 [0.09–0.43]	0.24 [0.08–0.54]	0.99
BASO (K/µL)	Median [Min.–Max]	0.06 [0.05–0.09]	0.06 [0.05–0.07]	0.06 [0.04–0.08]	0.06 [0.05–0.08]	0.11 [0.09–0.12]	0.06 [0.04–0.11]	0.09 [0.08–0.14]	0.08 [0.06–0.11]	0.06
FIBR (mg/dL)	Median [Min.–Max]	864.6 [694.58–1229.93]	1032 [790.09–1536.98]	1065 [712.95–1441.05]	769.5 [735.45–878.1]	795 [671.7–1077.08]	696.15 [617.25–1098.9]	761.4 [562.35–1033.2]	1111.8 [630.3–1388.33]	0.2
N/L ratio	Median[Min.–Max]	0.6 6[0.6–0.9]	0.85 [0.67–1.01]	0.99 [0.75–1.62]	0.55 [0.48–0.85]	0.91 [0.44–1.11]^b^	0.72 [0.56–1.32]^b^	1.12 [0.75–1.93]^a^	0.69 [0.46–1.07]^b^	0.02

Abbreviations**:** IT = individual treatment, M = metaphylactic treatment, M + IT = metaphylactic and individual treatment, NT = no treatment, RBC = Red blood cell, HGB = Haemoglobin, HTC = Haematocrit, MCV = Mean corpuscular volume, MCH = Mean corpuscular haemoglobin, MCHC = Mean corpuscular haemoglobin concentration, RDW = Red blood cell distribution width, PLT = Platelets, NEU = Neutrophils, WBC = white blood cells, MONO = Monocytes, LYM = Lymphocytes, EOS = Eosinophils, BASO = Basophils, FIBR = Fibrinogen, N/L ratio = Neutrophils: Lymphocytes ratio, M/µL= 10^6^ per microliter, %,=percentage, K/µL = 10^3^ per microliter, g/dL = grams per decilitre, fL = femtoliter, pg = picogram, mg/dL = milligram per decilitre, ^a,b,ab^, denote significant differences among the treatment protocol.

The effect of the different antimicrobial protocols at T0 on the clinical findings at T1 (respiratory disease, integument lesions, lameness, diarrhoea) are presented in Table 4. No statistically significant effect (*p*-value ≤ 0.05) was observed. The effect of the different antimicrobial protocols at T1 on the lung lesions observed at the abattoir are presented in Table 5. No statistically significant effect (*p*-value ≤ 0.05) was observed, too.

**Table 4.** Effect of different treatment protocols at T0 on the clinical findings at T1.

Clinical findings	Category	Days								*p*-value
		T0				T1				
		Treatment protocol				Treatment protocol				
		IT	M	M + IT	NT	IT	M	M + IT	NT	
Respiratory disease	N	21	45	93	17	21	69	66	24	0.55
	Y	8	9	49	7	8	26	37	13	
Integument alterations	N	36	54	150	24	19	52	59	16	0.31
	Y	0	0	0	0	10	43	44	21	
Lameness	N	36	54	150	24	28	94	101	37	0.59
	Y	0	0	0	0	1	1	2	0	
Diarrhea	N	36	53	149	24	28	94	101	37	0.59
	Y	0	1	1	0	1	1	2	0	

Abbreviations: N = No, Y = yes, IT = Individual treatment, M = Metaphylactic treatment, M + IT = Metaphylactic and individual treatment, NT = No Treatment.

**Table 5.** Effect of different treatment protocols at T1 on the lung lesions observed at the abattoir.

	Category	Days								*p*-value
		T0				T1				
		Treatment protocol				Treatment protocol				
		IT	M	M + IT	NT	IT	M	M + IT	NT	
Lung lesions	0	NA	NA	NA	NA	0	4	6	3	0.25
	1	NA	NA	NA	NA	13	23	49	11	
	2	NA	NA	NA	NA	7	11	36	4	
	3	NA	NA	NA	NA	1	0	4	0	

Abbreviations: 0 = No evidence of parenchymal alteration (healthy), 1= parenchymal inflammatory lesions in 1 to 25% of the lung (mild pneumonia), 2 = parenchymal inflammatory lesions in 25% to 50% of the lung (moderate pneumonia), 3= parenchymal inflammatory lesions in more than 50% of the lung (severe pneumonia), NA = Not applicable, IT = individual treatment, M = metaphylactic treatment, M + IT = metaphylactic and individual treatment, NT = no treatment.

### Effect of the anti-inflammatory treatment on blood analysis, clinical findings and lung at abattoir

The effect of NSAID treatment at T0 on blood analysis at T1 are presented in Table 6. At T1, there was a statistically significant effect (*p*-value ≤ 0.05) of the NSAID treatment on the WBC, neutrophils and N/L ratio. The WBC, neutrophils and N/L ratio of animals that were treated with an NSAID was significantly higher than that not treated with an NSAID. The effect of the NSAID treatment at T0 on the clinical findings at T1 (respiratory disease, integument lesions, lameness, diarrhoea) are presented in Table 7. No statistically significant effect (*p*-value ≤ 0.05) was observed.

**Table 6.** Effect of the non-steroid anti-inflammatory treatment in blood parameters at T1.

Blood parameters		Days				
		T0		T1		
		AI		AI		
		N	Y	N	Y	*p*-value
WBC (K/µL)	Median [Max.–Min]	8.3 [7.08–9.97]	8.79 [7.5–10.07]	9.89 [7.88–12.51]	10.54 [8.73–13.02]	0.05
NEU (K/µL)	Median [Max.–Min]	3.19 [254–3.97]	3.46 [2.81–4.01]	3.36 [2.64–4.39]	5.07 [3.02–6.92]	<.01
N/L ratio	Median [Max. –Min]	0.8 [0.58–1.27]	0.92 [0.67–1.36]	0.76 [0.53–1.25]	1.07 [0.76–1.93]	0.01

Abbreviations: NEU = Neutrophils, WBC = white blood cells, N/L ratio = Neutrophils/Lymphocytes ratio, K/µL = 10^3^ per microliter, AI = non-steroid anti-inflammatory treatment.

**Table 7.** Effect of the non-steroid anti-inflammatory treatment on the clinical findings at T1.

		Days				
		T0		T1		
		AI		AI		
Clinical findings	Category	N	Y	N	Y	*p*-value
Respiratory disease	N	99	77	107	73	0.47
	Y	30	43	46	38	
Integument alterations	N	NA	NA	79	67	0.16
	Y	NA	NA	74	44	
Lameness	N	NA	NA	151	109	0.75
	Y	NA	NA	2	2	
Diarrhea	N	NA	NA	151	109	0.75
	Y	NA	NA	2	2	

Abbreviations: NA = Not applicable, AI = non-steroid anti-inflammatory treatment, N = No, Y = Yes.

The effect of the NSAID treatment at T1 on the lung lesions observed at the abattoir are presented in Table 8. There was a statistically significant effect (*p*-value ≤ 0.05) of the NSAID treatment on the lung lesions observed at the abattoir.

**Table 8.** Effect of the non-steroid anti-inflammatory treatment on the lung lesions observed at the abattoir.

		Days				
		T0		T1		
		AI		AI		
	Category	N	Y	N	Y	*P*-value
Lung lesions	0	NA	NA	11	2	<.01
	1	NA	NA	56	40	
	2	NA	NA	25	33	
	3	NA	NA	0	5	

Abbreviations: 0 = no evidence of parenchymal alteration (healthy), 1 = parenchymal inflammatory lesions in 1 to 25% of the lung (mild pneumonia), 2 = parenchymal inflammatory lesions in 25% to 50% of the lung (moderate pneumonia), 3 = parenchymal inflammatory lesions in more than 50% of the lung (severe pneumonia), NA = Not applicable, AI = non-steroid anti-inflammatory treatment, N = No, Y = Yes.

## Discussion

This observational study dealt with the assessment of different BRD antimicrobial and NSAID use on health and welfare in newly introduced beef cattle in an intensive fattening system. The first period of the intensive cycle is widely reported to be the more stressful and more susceptible to illness status by young animals (EFSA Panel on Animal Health and Welfare (AHAW) 2012). To reduce this stress period an adaption diet is commonly provided (Cozzi 2007). In the present study the provided diet was adequate in terms of starch, protein, and fibrous fractions requirements (Fusaro et al. 2022; Fusaro et al. 2021; National Research Council (NRC) 2000). So, we believe that the obtained results are absents from bias due to dietary management.

In our study, for the welfare assessment we used a method based on a modified version of the Italian protocol for beef cattle welfare assessment that is included in the ClassyFarm system (Bertocchi et al. 2020). This method was applied at T0 and T1, in order to achieve consistency over a critical time. From T0 to T1, a decrease in welfare was noticed due to a reduction in Area C Animal-based indicators score. The observed decrease in Area C score might be associated with a stress response to both physical (i.e. transportation, new environment, new feed) and psychological (i.e. maternal separation, social mixing) stressors. Epidemiological research has related a large spectrum of stressors, in particular transportation, as factors contributing to higher disease susceptibility including BRD (Chen et al. 2015). Transportation causes a general immunosuppression that makes it possible for many opportunistic pathogens to invade the respiratory tract and result in BRD (Earley et al. 2017).

In our study, at arrival to the unit (T0) 34% of the animals already presented coughing and 48.86% nasal discharge, and more than 79% of the nasal swab pools for RT-PCR for *M. bovis* were positive indicating a high incidence of BRD at the moment of the introduction in the fattening unit. Moreover, at T1 a moderate increase in coughing (52.65%) and a slight decrease in nasal discharge (41.28%) were noticed, and more than 95% of the nasal swabs for culture of *M. bovis* tested positive. These findings support the increase in BRD incidence in the farm. We could speculate that a certain percentage of animals, although clinically healthy, started their transport to the fattening unit in Italy already infected or alternatively were exposed to pathogens during transportation. They then developed the diseases once in the new location in Italy. In fact, stress factors such as transportation and social and environmental change, might have a negative influence on the regulation of innate immunity (Chen et al. 2015). This could explain the occurrence of clinical disease already at arrival at the fattening unit in animals exposed to BRD-pathogens before transportation (Padalino et al. 2021; Cirone et al. 2019). Furthermore, these animals could be active shedders and infect other animals during transportation. Naive animals that are exposed both to pathogens and stress factors are more susceptible to develop the clinical disease (Castillo-Alcala et al. 2012). In addition, intensive systems of housing and rearing animals can also create favourable conditions for the occurrence of BRD (Catania et al. 2020). The housing structure, type and quality of flooring, microclimatic conditions, space allowances/pen size are conditions that may be factors influencing animal health (Cozzi et al. 2009).

In the case of *M. bovis*, Castillo-Alcala et al. (2012) showed an increased prevalence from the day of arrival up to day 15 after arrival similar to the current study. Several studies on the occurrence of BRD in beef cattle transported from France to Italy revealed an increase of the prevalence of BRD-related pathogens (including *M. bovis*) after arrival at the Italian fattening units (Catania et al. 2020; Padalino et al. 2021; Cirone et al. 2019). One of these studies reported that BRD-related pathogens increased from 16% to 82.8% four days after arrival at the fattening unit (Padalino et al. 2021).

Herein, the effects of the different antimicrobial (NT, M, IT and M + IT) and NSAID treatments for BRD on the blood analysis were investigated. For the antimicrobial treatments, the neutrophil count of the group M + IT was significantly higher than that of groups NT and M, and the lymphocyte count of M + IT was significantly lower than that of IT. In fact, animals included in the M + IT group were clinically affected by BRD. However, there was an absence of statistically significance difference of the clinical findings between M + IT and IT groups. This could be explained by the fact that also animals included in the group IT were clinically affected by BRD. Therefore, our findings suggest that there was no difference between a performing a M + IT or only IT. Moreover, it could be associated with inefficiency of the used antimicrobials. The number of antimicrobial treatments is linked with a higher probability of bacteria resistant to at least one antimicrobial. In addition, antimicrobial resistance in BRD is higher when using a combination of antimicrobials with different pharmacodynamics. These observations suggest that consideration should be given to antimicrobial pharmacodynamics when selecting drugs for retreatment of BRD (Coetzee et al. 2019). Choosing an ineffective antimicrobial for BRD poses serious risks to both animals and their owners in terms of welfare and financial implications. The decision-making process must take into account all relevant information to select the ‘optimal’ antimicrobial drug for a given situation, often including the results of bacterial culture and antimicrobial susceptibility testing (Lubbers and Turnidge 2015). More targeted and selective use of antibiotics in the livestock industry will be required in light of the emergence of antibiotic-resistant pneumonia in feedlot cattle (Earley et al. 2017). For the NSAID treatment, the WBC, neutrophils and N/L ratio of animals that were treated with an NSAID was significantly higher than that not treated. NSAID may have immunomodulatory effects and interfere with the function of neutrophils by increasing cellular immunity that, consequently, decreases the immune response (Curry et al. 2005). The effect of different antimicrobial and NSAID treatments at T0 on the retrieved clinical findings at T1 was also investigated and revealed the absence of a statistically significant effect. The first two weeks after the introduction of cattle in beef fattening units seem to be the most critical period for the development of BRD, even when metaphylactic treatment and vaccination are started (Pratelli et al. 2021). Furthermore, it has been reported that in feedlots, *M. bovis* can be resistant to most of the antimicrobials that are used to treat BRD (García-Galán et al. 2021; Jelinski et al. 2020). Herein, unfortunately, we did not investigate possible antimicrobial resistance. Furthermore, the clinical signs of BRD may not be detected at early stage of the disease and many animals may be undetected so that when detected the disease stage is advanced and the treatment success is less likely. It was suggested that the accuracy of current approaches for the early detection, prognosis, and diagnosis of BRD is still low, necessitating further study into BRD diagnostics (Chai et al. 2022).

We further observed that more than 90% of the lungs at the abattoir presented at least one lung lesion and the most prevalent category was mild pneumonia. Our findings show a very high prevalence of lung lesions when compared to previous reports (43–72%) (Caucci et al. 2018; Thompson et al. 2006; Wittum et al. 1996). Moreover, the effect of different antimicrobial protocols on the lung lesions at the abattoir showed absence of a statistically significant effect similar to Caucci et al. (2018). Chronic *M. bovis*-associated lung lesions may represent a dynamic situation of bacterial clearance and reinfection with genotypically different *M. bovis* strains. These findings could explain the ineffectiveness of the antimicrobial treatment for chronic pneumonia associated with *M. bovis* (Castillo-Alcala et al. 2012). *M. bovis* involvement in BRD can result in persistent pneumonia that does not respond well to antimicrobial therapy (García-Galán et al. 2021; Jelinski et al. 2020). Contrarily, a significant effect of the NSAID treatment on the lung lesions was observed at the abattoir. The lung lesions from the categories healthy and mild pneumonia were significantly lower in animals that received an anti-inflammatory treatment in the first 15 days after arrival to the farm. These findings suggest that an NSAID treatment for BRD may help to decrease lung inflammation. Compiani et al. (2020) reported that the use of NSAID in beef cattle at arrival to a fattening unit reduces the incidence of BRD.

## Conclusions

In summary, our observational study revealed a decrease in welfare during the first 15 days after arrival to the farm, in particular considering the score in Area C animal-based indicators. Our findings indicate that the prevalence of BRD, most likely associated with *M. bovis*, in this beef cattle population was already high at the time of arrival to the farm and increased during the first 15 days after arrival. We further observed an absence of association between different antimicrobial protocols (IT, M, M + IT, NT) started at arrival and the retrieved clinical findings at 15 days after arrival. Moreover, we observed a high prevalence of lung lesions at the abattoir. An absence of association between different antimicrobial protocols (IT, M, M + IT, NT) administered in the first 15 days after arrival and the lung lesions observed at the abattoir was also noticed. In contrast, an association between NSAID treatment and lung lesions was noticed indicating that NSAID treatments for BRD may help to the decrease lung inflammation. Our findings indicate that BRD was a major welfare and health problem in the studied population. Indeed, our findings evidence the difficulties of antimicrobial treatment and the potential efficiency of NSAID treatment of *M. bovis* BRD-associated pneumonia. Therefore, enhancing farming practices, animal health and welfare should primarily be considered to reduce disease prevalence and antimicrobial usage. Furthermore, the use of NSAIDs could represent an optional approach to control BRD and reduce antimicrobial usage but more research should be performed to validate this hypothesis. Our observational study highlights the real challenge in the management of BRD conditions in intensive fattening systems.

## Supplementary Material

Supplemental Material
