# Supplementary material for: Evaluation of antimicrobial and non-steroidal anti-inflammatory treatments for BRD on health and welfare in fattening bulls: a cross-sectional study
Source: Vet Q. 2024 May 6;44(1):1–11. doi: 10.1080/01652176.2024.2347928 (PMC11078067; doi:10.1080/01652176.2024.2347928)
Supplement: Supplemental Material [file TVEQ_A_2347928_SM0898.zip › Supplementary figure S1.pdf]

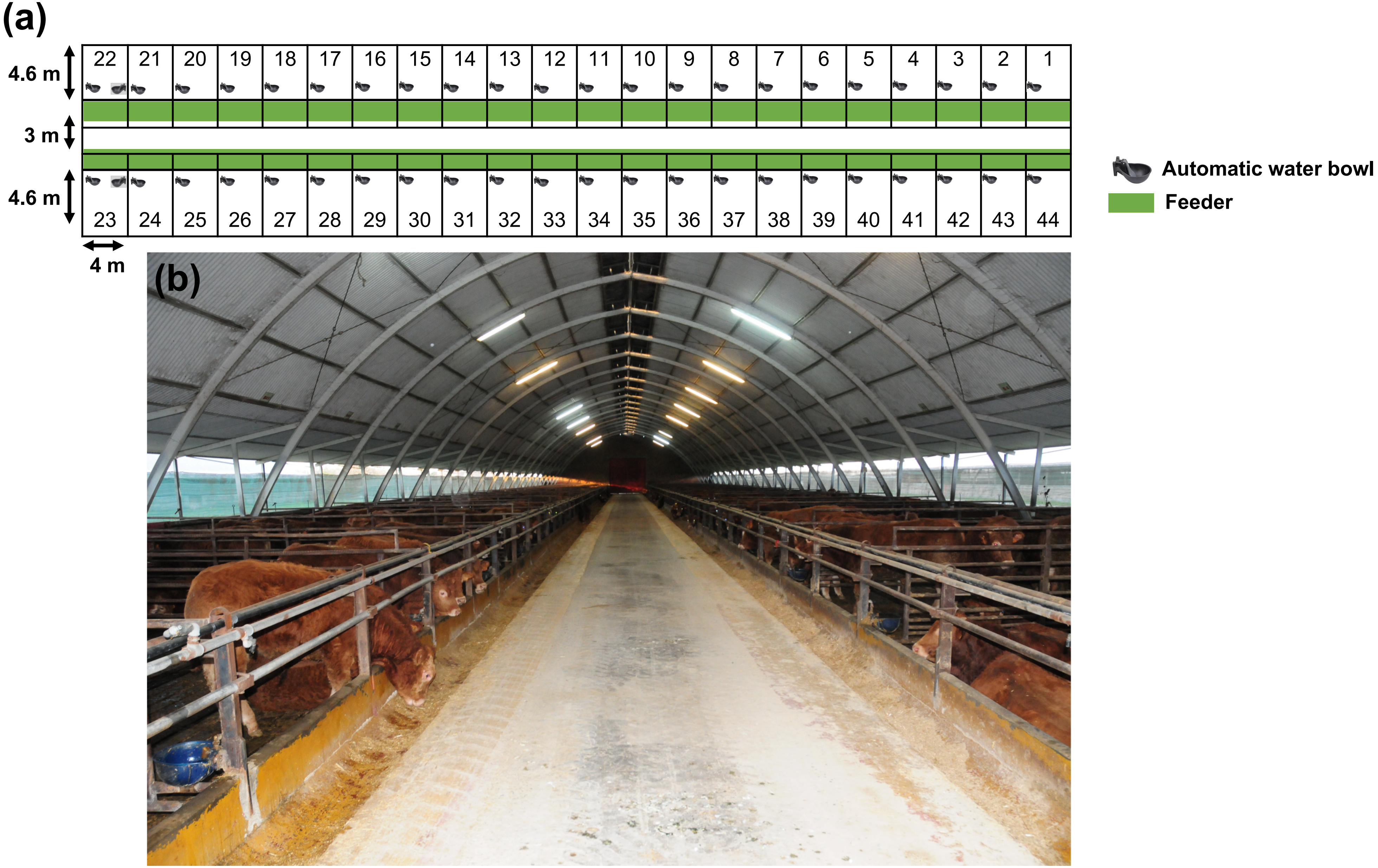

Supplementary Figure S1. Structure of the commercial fattening unit. **(a)** Schematic representation of the structure of the commercial fattening unit, position of feeders and automatic water bowls, dimension of the pens and corridors. **(b)** Image of the commercial fattening unit.
