# Supplementary material for: Evaluation of antimicrobial and non-steroidal anti-inflammatory treatments for BRD on health and welfare in fattening bulls: a cross-sectional study
Source: Vet Q. 2024 May 6;44(1):1–11. doi: 10.1080/01652176.2024.2347928 (PMC11078067; doi:10.1080/01652176.2024.2347928)
Supplement: Supplemental Material [file TVEQ_A_2347928_SM0898.zip › Supplementary figure S2.pdf]

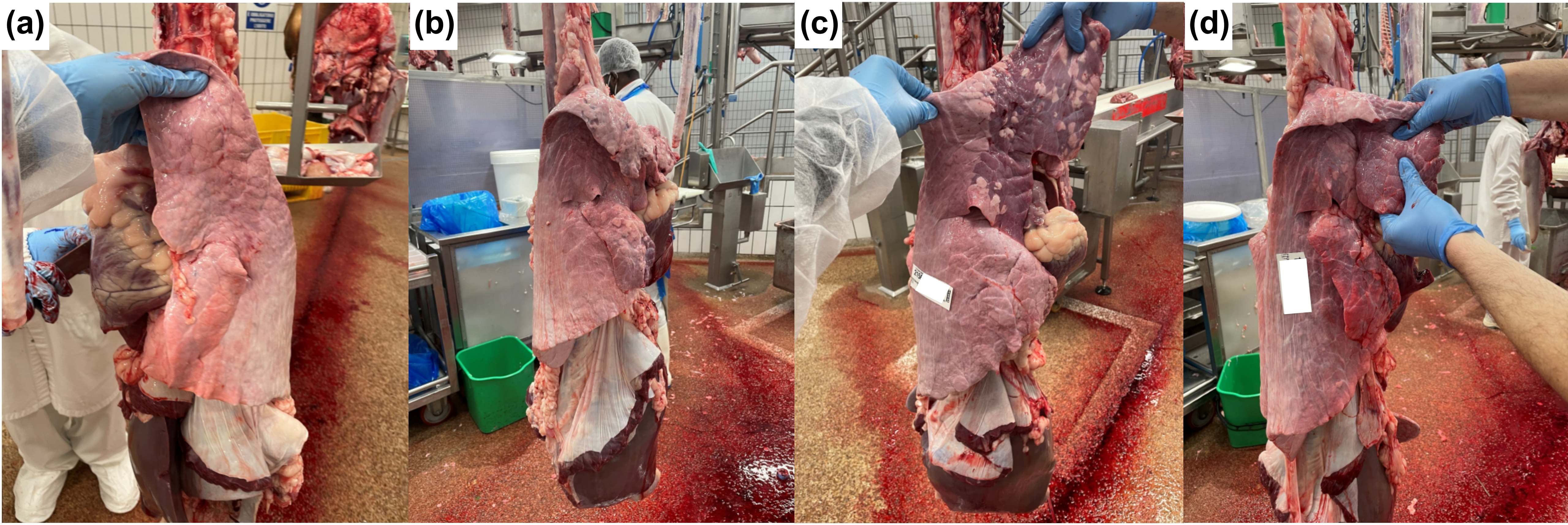

Supplementary Figure S2. Lung score based on an estimation of the extension of diseased parenchyma. **(a)** no evidence of parenchymal alteration (healthy); **(b)** parenchymal inflammatory lesions in 1 to 25% of the lung (mild pneumonia); **(c)** parenchymal inflammatory lesions in 25% to 50% of the lung (moderate pneumonia); **(d)** parenchymal inflammatory lesions in more than 50% of the lung (severe pneumonia).
