# Supplementary material for: Evaluation of antimicrobial and non-steroidal anti-inflammatory treatments for BRD on health and welfare in fattening bulls: a cross-sectional study
Source: Vet Q. 2024 May 6;44(1):1–11. doi: 10.1080/01652176.2024.2347928 (PMC11078067; doi:10.1080/01652176.2024.2347928)
Supplement: Supplemental Material [file TVEQ_A_2347928_SM0898.zip › Supplementary Table S1.pdf]

Supplementary Table S1. Analysis of the adaptation TMR diet (T0 and T1) and chemical analysis.

TMR	Feed, kg af
Wheat silage	3.5
Meadow hay[1]	1.2
Wheat straw	1.1
Beat pulp	1.3
Corn, finely ground[2]	1.1
Soybean meal	0.5
Cane molasses[3]	0.5
Min&Vit Premix	0.3
	**Nutrients, %DM**
DM	70.07
UFC	0.81
CP[4]	11.25
Ash	8.78
EE[5]	2.06
Starch	13.57
Sugars	7.37
NDF[6]	39.21
ADF[7]	25.79
ADL[8]	3.45

[1] the quality of the hay was checked to ensure the absence of molds and spores (Cavallini et al. 2022). [2] the corn was below the EU maxim tolerable level (Girolami et al. 2022). [3] molasses were properly characterized (Palmonari et la. 2021). [4] Crude protein. [5] ether extract. [6] neutral detergent fiber. [7] acid detergent fiber. [8] acid detergent lignin.

Cavallini D, Penazzi L, Valle E, Raspa F, Bergero D, Formigoni A, Fusaro I. 2022. When changing the hay makes a difference: A series of case reports. J Equine Vet Sci. 113:103940.

Girolami F, Barbarossa A, Badino P, Ghadiri S, Cavallini D, Zaghini A, Nebbia C. 2022. Effects of turmeric powder on aflatoxin M1 and aflatoxicol excretion in milk from dairy cows exposed to aflatoxin b1 at the EU maximum tolerable levels. Toxins 14: 430.

Palmonari A, Cavallini D, Sniffen CJ, Fernandes L, Holder P, Fusaro I, Giammarco M, Formigoni A, Mammi LME. 2021. In vitro evaluation of sugar digestibility in molasses. Ital. J. Anim. Sci. 20:571-577.
