# Supplementary material for: Evaluation of antimicrobial and non-steroidal anti-inflammatory treatments for BRD on health and welfare in fattening bulls: a cross-sectional study
Source: Vet Q. 2024 May 6;44(1):1–11. doi: 10.1080/01652176.2024.2347928 (PMC11078067; doi:10.1080/01652176.2024.2347928)
Supplement: Supplemental Material [file TVEQ_A_2347928_SM0898.zip › Supplementary Table S2.pdf]

Supplementary Table S2. Schematic table used for recording the inspective clinical examination per pen.

Pen Number ________________ **Date** ____

Ear Tag	MS [1]	BCS [2]	CS [3]	SL	LS [4]	RF [5]	N cough [6]	ND [7]	OD [8]	Other
		1 6<br>2 7<br>3 8<br>4 9<br>5	0<br>1<br>2		0<br>1<br>2<br>3			• Present/Absent<br>• Bilateral/Monolateral<br>• Mucous/Hemorrhagic/ Purulent	• Present/Absent<br>• Bilateral/Monolateral<br>• Mucous/Hemorrhagic/ Purulent	
		1 6<br>2 7<br>3 8<br>4 9<br>5	0<br>1<br>2		1<br>2<br>3<br>4			• Present/Absent<br>• Bilateral/Monolateral<br>• Mucous/Hemorrhagic/ Purulent	• Present/Absent<br>• Bilateral/Monolateral<br>• Mucous/Hemorrhagic/ Purulent	
		1 6<br>2 7<br>3 8<br>4 9<br>5	0<br>1<br>2		1<br>2<br>3<br>4			• Present/Absent<br>• Bilateral/Monolateral<br>• Mucous/Hemorrhagic/ Purulent	• Present/Absent<br>• Bilateral/Monolateral<br>• Mucous/Hemorrhagic/ Purulent	
		1 6<br>2 7<br>3 8<br>4 9<br>5	0<br>1<br>2		1<br>2<br>3<br>4			• Present/Absent<br>• Bilateral/Monolateral<br>• Mucous/Hemorrhagic/ Purulent	• Present/Absent<br>• Bilateral/Monolateral<br>• Mucous/Hemorrhagic/ Purulent	
		1 6<br>2 7<br>3 8<br>4 9<br>5	0<br>1<br>2		1<br>2<br>3<br>4			• Present/Absent<br>• Bilateral/Monolateral<br>• Mucous/Hemorrhagic/ Purulent	• Present/Absent<br>• Bilateral/Monolateral<br>• Mucous/Hemorrhagic/ Purulent	
		1 6<br>2 7<br>3 8<br>4 9<br>5	0<br>1<br>2		1<br>2<br>3<br>4			• Present/Absent<br>• Bilateral/Monolateral<br>• Mucous/Hemorrhagic/ Purulent	• Present/Absent<br>• Bilateral/Monolateral<br>• Mucous/Hemorrhagic/ Purulent	

Abbreviations: MS=Mental status; BCS=Body condition score; CS=cleanness scoring; SL=Skin lesions; LS=Locomotion scoring; RF=respiratory findings; ND=nasal discharge; OD=ocular discharge.

[1] the following nomenclatures were used to describe the mental status: alert, dullness, stupor, coma (Lorenz et al., 2011)

[2] based on the Guide to Body Condition Scoring Beef Cows and Bulls, Kansas State University (Farney et al., 2016).

[3] has in consideration the cleanness of flanks including tail and lower hindlimb; score 0 = no dirt or only minor fresh or dried splashing, score 1= an area of dirtiness at least palm size (10 x 15cm), score 2= an area of dirtiness amounting to at least forearm length (40cm) in any dimension; scoring system adapted from AHDB available from https://projectblue.blob.core.windows.net/media/Default/Imported%20Publication%20Docs/Cleanliness%20scorecard%20optimal%20dairy%20systems.pdf

[4] based on Zinpro Step-Up Beef Cattle Locomotion Scoring System

[5] Including the respiratory pattern and respiratory frequency (Baumgartner and Wittek, 2017).

[6] Number of spontaneous coughs in an interval of 10 minutes.

[7] the type of nasal discharge was classified as following: absent or present; if present monolateral or bilateral; mucous/hemorrhagic/purulent.

[8] the type of ocular discharge was classified as following: absent or present; if present monolateral or bilateral; mucous or purulent.
