# Supplementary material for: Evaluation of antimicrobial and non-steroidal anti-inflammatory treatments for BRD on health and welfare in fattening bulls: a cross-sectional study
Source: Vet Q. 2024 May 6;44(1):1–11. doi: 10.1080/01652176.2024.2347928 (PMC11078067; doi:10.1080/01652176.2024.2347928)
Supplement: Supplemental Material [file TVEQ_A_2347928_SM0898.zip › Supplementary Table S3.pdf]

Supplementary Table S3. Different antimicrobial drugs used for BRD treatment in the fattening unit.

ID	Treatment	Antimicrobial (commercial name and active principle)
1	M+IT	Zuprevo18%® (Tildipirosin)
2	NT	
3	M	
4	M+IT	Nuflor® (Florfenicol), Zuprevo18%® (Tildipirosin)
5	NT	
6	NT	
7	NT	
8	NT	
9	M	
10	M	
11	M	
12	M	
13	M	
14	NT	
15	IT	Nuflor® (Florfenicol)
16	M	
17	M	
18	M	
19	M	
20	M	
21	M	
22	NT	
23	M	
24	M	
25	M+IT	Nuflor® (Florfenicol)
26	M+IT	Nuflor® (Florfenicol)
27	M	
28	M+IT	Nuflor® (Florfenicol)
29	M	
30	M	
31	M	
32	M	
33	M+IT	Nuflor® (Florfenicol)
34	M+IT	Zuprevo18%® (Tildipirosin),Depocillina® (Penicillin G procaine),Valemas10® (Enrofloxacina),Nuflor® (Florfenicol)
35	M	
36	M	
37	M	

38	M	
39	M+IT	Nuflor® (Florfenicol)
40	M	
41	M+IT	Nuflor® (Florfenicol),
42	M	
43	M	
44	M	
45	M	
46	M	
47	M+IT	Nuflor® (Florfenicol),
48	NT	
49	M	
50	M+IT	Nuflor® (Florfenicol),
51	M+IT	Nuflor® (Florfenicol),
52	M	
53	M	
54	M	
55	M	
56	M	
57	M	
58	NT	
59	NT	
60	M+IT	Nuflor® (Florfenicol),Zuprevo18%® (Tildipirosin)
61	M+IT	Zuprevo18%® (Tildipirosin)
62	NT	
63	M+IT	Zuprevo18%® (Tildipirosin)
64	M+IT	Nuflor® (Florfenicol),Zuprevo18%® (Tildipirosin)
65	M+IT	Zuprevo18%® (Tildipirosin)
66	M+IT	Zuprevo18%® (Tildipirosin)
67	M+IT	Zuprevo18%® (Tildipirosin)
68	M+IT	Nuflor® (Florfenicol),Zuprevo18%® (Tildipirosin)
69	NT	
70	M+IT	Nuflor® (Florfenicol),Zuprevo18%® (Tildipirosin)
71	M+IT	Nuflor® (Florfenicol),Zuprevo18%® (Tildipirosin)
72	M+IT	Nuflor® (Florfenicol),Zuprevo18%® (Tildipirosin)
73	M	
74	M	
75	NT	
76	M	
77	NT	
78	NT	

79	M+IT	Nuflor® (Florfenicol)
80	M	
81	M	
82	M+IT	Nuflor® (Florfenicol)
83	M+IT	Nuflor® (Florfenicol)
84	NT	
85	IT	Nuflor® (Florfenicol)
86	M+IT	Zuprevo18%® (Tildipirosin)
87	M+IT	Zuprevo18%® (Tildipirosin)
88	M	
89	M+IT	Nuflor® (Florfenicol)
90	M+IT	Nuflor® (Florfenicol)
91	M	
92	M	
93	M	
94	M	
95	IT	Nuflor® (Florfenicol)
96	NT	
97	IT	Nuflor® (Florfenicol)
98	NT	
99	NT	
100	M	
101	M+IT	Zuprevo18%® (Tildipirosin)
102	M+IT	Zuprevo18%® (Tildipirosin)
103	M+IT	Zuprevo18%® (Tildipirosin)
10	M+IT	Nuflor® (Florfenicol),Zuprevo18%® (Tildipirosin)
105	M+IT	Nuflor® (Florfenicol),Zuprevo18%® (Tildipirosin)
106	M+IT	Nuflor® (Florfenicol),Zuprevo18%® (Tildipirosin),Forcyl® (Marbofloxacin)
107	M+IT	Zuprevo18%® (Tildipirosin)
108	M+IT	Nuflor® (Florfenicol),Zuprevo18%® (Tildipirosin)
109	IT	Nuflor® (Florfenicol),Zuprevo18%® (Tildipirosin)
110	IT	Nuflor® (Florfenicol),Zuprevo18%® (Tildipirosin)
111	IT	Zuprevo18%® (Tildipirosin)
112	IT	Nuflor® (Florfenicol),Zuprevo18%® (Tildipirosin)
113	M+IT	Nuflor® (Florfenicol),Zuprevo18%® (Tildipirosin)
114	M+IT	Nuflor ® (Florfenicol),Zuprevo18%® (Tildipirosin)
115	M+IT	Zuprevo18%® (Tildipirosin)
116	IT	Nuflor® (Florfenicol),Zuprevo18%® (Tildipirosin)
117	IT	Nuflor® (Florfenicol),Zuprevo18%® (Tildipirosin)
118	IT	Nuflor® (Florfenicol),Zuprevo18%® (Tildipirosin), Vetamplius® (Ampicillin)

119	IT	Nuflor® (Florfenicol),Zuprevo18%® (Tildipirosin)
120	IT	Zuprevo18%® (Tildipirosin)
121	IT	Zuprevo18%® (Tildipirosin), Vetamplius® (Ampicillin)
122	IT	Nuflor® (Florfenicol),Zuprevo18%® (Tildipirosin)
123	M+IT	Zuprevo18%® (Tildipirosin)
124	M	
125	IT	Nuflor® (Florfenicol)
126	NT	
127	M	
128	NT	
129	M+IT	Nuflor® (Florfenicol),Zuprevo18%® (Tildipirosin), Longocillina® (Amoxicillin Trihydrate)
130	M+IT	Nuflor® (Florfenicol),Zuprevo18%® (Tildipirosin)
131	M+IT	Nuflor® (Florfenicol),Zuprevo18%® (Tildipirosin)
132	M+IT	Nuflor® (Florfenicol)
133	M+IT	Nuflor® (Florfenicol),Zuprevo18%® (Tildipirosin)
134	M+IT	Zuprevo18%® (Tildipirosin)
135	M+IT	Zuprevo18%® (Tildipirosin)
136	M+IT	Zuprevo18%® (Tildipirosin)
137	M+IT	Nuflor® (Florfenicol),Zuprevo18%® (Tildipirosin)
138	M+IT	Nuflor® (Florfenicol),Zuprevo18%® (Tildipirosin)
139	M+IT	Nuflor® (Florfenicol),Zuprevo18%® (Tildipirosin)
140	M+IT	Zuprevo18%® (Tildipirosin)
141	M+IT	Zuprevo18%® (Tildipirosin)
142	M+IT	Zuprevo18%® (Tildipirosin)
143	M+IT	Nuflor® (Florfenicol),Zuprevo18%® (Tildipirosin)
144	M+IT	Zuprevo18%® (Tildipirosin)
145	M+IT	Zuprevo18%® (Tildipirosin)
146	M+IT	Zuprevo18%® (Tildipirosin)
147	M+IT	Nuflor® (Florfenicol),Zuprevo18%® (Tildipirosin)
148	M+IT	Zuprevo18%® (Tildipirosin)
149	M+IT	Zuprevo18%® (Tildipirosin),Longocillina® (Amoxicillin Trihydrate)
150	M+IT	Zuprevo18%® (Tildipirosin)
151	M+IT	Nuflor® (Florfenicol),Zuprevo18%® (Tildipirosin)
152	M+IT	Zuprevo18%® (Tildipirosin)
153	M+IT	Nuflor® (Florfenicol),Zuprevo18%® (Tildipirosin)
154	M+IT	Zuprevo18%® (Tildipirosin)
155	M+IT	Zuprevo18%® (Tildipirosin)
156	M+IT	Zuprevo18%® (Tildipirosin)
157	M+IT	Zuprevo18%® (Tildipirosin)
158	M+IT	Zuprevo18%® (Tildipirosin)

159	M+IT	Zuprevo18%® (Tildipirosin)
160	M+IT	Zuprevo18%® (Tildipirosin)
161	M+IT	Nuflor® (Florfenicol),Zuprevo18%® (Tildipirosin)
162	M+IT	Zuprevo18%® (Tildipirosin)
163	M+IT	Nuflor® (Florfenicol),Zuprevo18%® (Tildipirosin)
164	M+IT	Nuflor® (Florfenicol),Zuprevo18%® (Tildipirosin)
165	M+IT	Nuflor® (Florfenicol),Zuprevo18%® (Tildipirosin)
166	IT	Zuprevo18%® (Tildipirosin)
167	M+IT	Nuflor® (Florfenicol),Zuprevo18%® (Tildipirosin)
168	M+IT	Zuprevo18%® (Tildipirosin)
169	M+IT	Zuprevo18%® (Tildipirosin)
170	M+IT	Zuprevo18%® (Tildipirosin)
171	M+IT	Zuprevo18%® (Tildipirosin)
172	M+IT	Zuprevo18%® (Tildipirosin)
173	M+IT	Zuprevo18%® (Tildipirosin)
174	M+IT	Zuprevo18%® (Tildipirosin)
175	IT	Zuprevo18%® (Tildipirosin)
176	M+IT	Nuflor® (Florfenicol),Zuprevo18%® (Tildipirosin)
177	M+IT	Zuprevo18%® (Tildipirosin)
178	M+IT	Nuflor® (Florfenicol),Zuprevo18%® (Tildipirosin)
179	M+IT	Zuprevo18%® (Tildipirosin)
180	M+IT	Zuprevo18%® (Tildipirosin)
181	M+IT	Zuprevo18%® (Tildipirosin)
182	M+IT	Zuprevo18%® (Tildipirosin)
183	M+IT	Nuflor® (Florfenicol), Zuprevo18%® (Tildipirosin)
184	M+IT	Zuprevo18%® (Tildipirosin)
185	M+IT	Zuprevo18%® (Tildipirosin)
186	M+IT	Nuflor® (Florfenicol),Zuprevo18%® (Tildipirosin)
187	IT	Zuprevo18%® (Tildipirosin)
188	IT	Zuprevo18%® (Tildipirosin)
189	IT	Nuflor® (Florfenicol),Zuprevo18%® (Tildipirosin)
190	IT	Nuflor® (Florfenicol),Zuprevo18%® (Tildipirosin)
191	IT	Zuprevo18%® (Tildipirosin)
192	IT	Zuprevo18%® (Tildipirosin)
193	M+IT	Nuflor® (Florfenicol),Zuprevo18%® (Tildipirosin)
194	M+IT	Depocillina® (Penicillin G procaine)
195	M+IT	Bimoxylla (Amoxicillin),Nuflor® (Florfenicol),Zuprevo18%® (Tildipirosin)
196	M+IT	Zuprevo18%® (Tildipirosin)
197	IT	Nuflor® (Florfenicol)
198	M+IT	Zuprevo18%® (Tildipirosin)

199	M+IT	Longocillina® (Amoxicillin Trihydrate),Nuflor®(Florfenicol),Zuprevo18%® (Tildipirosin)
200	M+IT	Nuflor® (Florfenicol), Zuprevo18%® (Tildipirosin)
201	M+IT	Nuflor® (Florfenicol),Zuprevo18%® (Tildipirosin)
202	M+IT	Nuflor® (Florfenicol)
203	M	
204	IT	Longocillina® (Amoxicillin Trihydrate), Nuflor® (Florfenicol)
205	IT	Nuflor® (Florfenicol)
206	M+IT	Nuflor® (Florfenicol)
207	M+IT	Nuflor® (Florfenicol),Zuprevo18%® (Tildipirosin)
208	M+IT	Nuflor® (Florfenicol),Zuprevo18%® (Tildipirosin)
209	M+IT	Bimoxylla (Amoxicillin),Zuprevo18%® (Tildipirosin)
210	M+IT	Zuprevo18%® (Tildipirosin)
211	M+IT	Zuprevo18%® (Tildipirosin)
212	M+IT	Forcyl (Marbofloxacin),Nuflor® (Florfenicol),Zuprevo18%® (Tildipirosin)
213	M+IT	Nuflor® (Florfenicol),Zuprevo18%® (Tildipirosin)
214	M+IT	Nuflor® (Florfenicol),Zuprevo18%® (Tildipirosin)
215	M+IT	Nuflor® (Florfenicol),Zuprevo18%® (Tildipirosin)
216	M+IT	Zuprevo18%® (Tildipirosin)
217	M+IT	Nuflor® (Florfenicol),Zuprevo18%® (Tildipirosin)
218	M+IT	Vetamplius®(Ampicillin), Zuprevo18%® (Tildipirosin), Forcyl (Marbofloxacin), Longocillina® (Amoxicillin Trihydrate)
219	IT	Zuprevo18%® (Tildipirosin), Depocillina® (Z) (Penicillin G procaine)
220	IT	Nuflor®(Florfenicol),Zuprevo18%® (Tildipirosin)
221	M+IT	Nuflor®(Florfenicol),Zuprevo18%® (Tildipirosin)
222	M+IT	Nuflor®(Florfenicol),Zuprevo18%® (Tildipirosin)
223	M+IT	Nuflor®(Florfenicol),Zuprevo18%® (Tildipirosin), Longocillina® (Amoxicillin Trihydrate)
224	M+IT	Vetamplius®(Ampicillin), Zuprevo18%® (Tildipirosin)
225	M+IT	Zuprevo18%® (Tildipirosin)
226	M+IT	Zuprevo18%® (Tildipirosin)
227	M+IT	Zuprevo18%® (Tildipirosin)
228	IT	Nuflor® (Florfenicol),Zuprevo18%® (Tildipirosin)
229	IT	Zuprevo18%® (Tildipirosin)
230	M	
231	M	
232	NT	
233	NT	
234	NT	
235	M	
236	M	
237	M+IT	Nuflor® (Florfenicol)

238	M	
239	M+IT	Nuflor® (Florfenicol)
240	M+IT	Nuflor® (Florfenicol)
242	IT	Nuflor® (Florfenicol)
242	IT	Nuflor® (Florfenicol)
243	IT	Bimoxylla® (Amoxicillin), Nuflor® (Florfenicol),Zuprevo18%® (Tildipirosin), Vetamplius®(Ampicillin),Valemas10® (Enrofloxacina)
244	M+IT	Longocillina® (Amoxicillin Trihydrate), Zuprevo18%® (Tildipirosin), Nuflor® (Florfenicol)
245	M+IT	Longocillina® (Amoxicillin Trihydrate), Zuprevo18%® (Tildipirosin)
246	M+IT	Nuflor® (Florfenicol),Zuprevo18%® (Tildipirosin)
247	M+IT	Depocillina® (Penicillin G procaine), Valemas10® (Enrofloxacina), Zuprevo18%® (Tildipirosin)
248	M+IT	Nuflor® (Florfenicol),Zuprevo18%® (Tildipirosin)
249	M+IT	Zuprevo18%® (Tildipirosin)
250	M+IT	Forcyl® (Marbofloxacin), Longocillina® (Amoxicillin Trihydrate), Nuflor®(Florfenicol),Zuprevo18%® (Tildipirosin)
251	M+IT	Nuflor® (Florfenicol),Zuprevo18%® (Tildipirosin)
252	M+IT	Nuflor® (Florfenicol),Zuprevo18%® (Tildipirosin)
253	M+IT	Zuprevo18%® (Tildipirosin)
254	M+IT	Zuprevo18%® (Tildipirosin)
255	M+IT	Nuflor® (Florfenicol),Zuprevo18%® (Tildipirosin)
256	M+IT	Nuflor® (Florfenicol),Zuprevo18%® (Tildipirosin)
257	M+IT	Zuprevo18%® (Tildipirosin)
258	M+IT	Zuprevo18%® (Tildipirosin)
259	M+IT	Nuflor® (Florfenicol),Zuprevo18%® (Tildipirosin)
260	M+IT	Zuprevo18%® (Tildipirosin)
261	M+IT	Zuprevo18%® (Tildipirosin)
262	M+IT	Zuprevo18%® (Tildipirosin)
263	M+IT	Zuprevo18%® (Tildipirosin)
264	M+IT	Zuprevo18%® (Tildipirosin)

Abbreviations: ID=animal identification, IT= individual treatment M=metaphylactic treatment, M+IT=metaphylactic and individual treatment, NT=no treatment
