# Supplementary material for: Evaluation of antimicrobial and non-steroidal anti-inflammatory treatments for BRD on health and welfare in fattening bulls: a cross-sectional study
Source: Vet Q. 2024 May 6;44(1):1–11. doi: 10.1080/01652176.2024.2347928 (PMC11078067; doi:10.1080/01652176.2024.2347928)
Supplement: Supplemental Material [file TVEQ_A_2347928_SM0898.zip › Supplementary Table S4.pdf]

Supplementary Table S4. Different non-steroid anti-inflammatory drugs used for BRD treatment in the fattening unit.

ID	NSAID treatment at T0 (commercial name and active principle)	NSAID treatment at T0 (commercial name and active principle)
1		
2		
3		
4	Metacam® (Meloxicam)	Finadyne® (Flunixin meglumina), Salicil Vet® (Acetylsalicylic acid)
5		Metacam® (Meloxicam)
6		
7		
8		
9		
10		Metacam® (Meloxicam)
11		
12		
13		
14		
15	Finadyne® (Flunixin meglumina)	Finadyne® (Flunixin meglumina)
16		
17		
18		
19		
20		
21		
22		
23		
24		
25	Finadyne® (Flunixin meglumina)	
26	Finadyne® (Flunixin meglumina)	Finadyne® (Flunixin meglumina)
27		Metacam® (Meloxicam)
28	Metacam® (Meloxicam)	Finadyne® (Flunixin meglumina)
29		
30		
31		
32		
33	Finadyne® (Flunixin meglumina)	Finadyne® (Flunixin meglumina)
34	Finadyne® (Flunixin meglumina)	Dinalgen® (Ketoprofen), Salicil Vet® (Acetylsalicylic acid)
35		
36		

Metacam® (Meloxicam)	
Finadyne® (Flunixin meglumina)	
	Finadyne® (Flunixin meglumina)
Metacam® (Meloxicam)	Finadyne® (Flunixin meglumina)
	Finadyne® (Flunixin meglumina)
Metacam® (Meloxicam)	
Metacam® (Meloxicam)	
	Metacam® (Meloxicam)
Metacam® (Meloxicam)	
Metacam® (Meloxicam)	
	Finadyne® (Flunixin meglumina)
Metacam® (Meloxicam)	
Metacam® (Meloxicam)	
Metacam® (Meloxicam)	
Metacam® (Meloxicam)	

78		
79	Metacam® (Meloxicam)	Finadyne® (Flunixin meglumina)
80		
81		
82	Metacam® (Meloxicam)	
83	Metacam® (Meloxicam)	Finadyne® (Flunixin meglumina), Metacam®(Meloxicam)
84		
85	Finadyne® (Flunixin meglumina)	
86		
87		
88		
89	Metacam® (Meloxicam)	
90	Metacam® (Meloxicam)	
91		
92		
93		
94		
95	Metacam® (Meloxicam)	Metacam® (Meloxicam)
96		
97	Metacam® (Meloxicam)	Finadyne® (Flunixin meglumina)
98		
99		
100	Finadyne® (Flunixin meglumina)	
101		
102	Metacam® (Meloxicam)	
103		
10	Metacam® (Meloxicam)	
105	Metacam® (Meloxicam)	
106	Finadyne® (Flunixin meglumina), Metacam®(Meloxicam)	Finadyne® (Flunixin meglumina)
107		
108	Metacam® (Meloxicam)	
109	Metacam® (Meloxicam)	Finadyne® (Flunixin meglumina)
110	Metacam® (Meloxicam)	Finadyne® (Flunixin meglumina)
111		Finadyne® (Flunixin meglumina)
112	Metacam® (Meloxicam)	Finadyne® (Flunixin meglumina)
113	Metacam® (Meloxicam)	Salicil Vet® (Acetylsalicylic acid)
114	Metacam® (Meloxicam)	Salicil Vet® (Acetylsalicylic acid)
115		Metacam®(Meloxicam), Salicil Vet® (Acetylsalicylic acid)
116	Metacam® (Meloxicam)	Finadyne® (Flunixin meglumina), Metacam®(Meloxicam)

117	Metacam® (Meloxicam)	Finadyne® (Flunixin meglumina)
118	Metacam® (Meloxicam)	Finadyne® (Flunixin meglumina)
119	Metacam® (Meloxicam)	Finadyne® (Flunixin meglumina)
120		Finadyne® (Flunixin meglumina)
121		Finadyne® (Flunixin meglumina)
122	Metacam® (Meloxicam)	Finadyne® (Flunixin meglumina)
123		Salicil Vet® (Acetylsalicylic acid)
124		Finadyne® (Flunixin meglumina), Salicil Vet® (Acetylsalicylic acid)
125	Metacam® (Meloxicam)	
126		
127	Finadyne® (Flunixin meglumina)	
128		
129	Finadyne® (Flunixin meglumina), Metacam®(Meloxicam)	Dinalgen® (Ketoprofen), Salicil Vet® (Acetylsalicylic acid)
130	Metacam® (Meloxicam)	Salicil Vet® (Acetylsalicylic acid)
131	Metacam® (Meloxicam)	Salicil Vet® (Acetylsalicylic acid)
132	Metacam® (Meloxicam)	Salicil Vet® (Acetylsalicylic acid)
133	Metacam® (Meloxicam)	Salicil Vet® (Acetylsalicylic acid)
134		Salicil Vet® (Acetylsalicylic acid)
135		Salicil Vet® (Acetylsalicylic acid)
136		Salicil Vet® (Acetylsalicylic acid)
137	Metacam® (Meloxicam)	Salicil Vet® (Acetylsalicylic acid)
138	Finadyne® (Flunixin meglumina)	Finadyne® (Flunixin meglumina), Salicil Vet® (Acetylsalicylic acid)
139	Finadyne® (Flunixin meglumina)	Dinalgen® (Ketoprofen), Salicil Vet® (Acetylsalicylic acid)
140		Salicil Vet® (Acetylsalicylic acid)
141		Salicil Vet® (Acetylsalicylic acid)
142		Salicil Vet® (Acetylsalicylic acid)
143	Metacam® (Meloxicam)	Salicil Vet® (Acetylsalicylic acid)
144		Salicil Vet® (Acetylsalicylic acid)
145		Salicil Vet® (Acetylsalicylic acid)
146		Salicil Vet® (Acetylsalicylic acid)
147	Metacam® (Meloxicam)	Salicil Vet® (Acetylsalicylic acid)
148		Salicil Vet® (Acetylsalicylic acid)
149		Finadine, Salicil Vet® (Acetylsalicylic acid)
150		Dinalgen® (Ketoprofen), Salicil Vet® (Acetylsalicylic acid)
151	Metacam® (Meloxicam)	Salicil Vet® (Acetylsalicylic acid)
152		Salicil Vet® (Acetylsalicylic acid)
153	Metacam® (Meloxicam)	Salicil Vet® (Acetylsalicylic acid)
154		Salicil Vet® (Acetylsalicylic acid)

155		Salicil Vet® (Acetylsalicylic acid)
156		Salicil Vet® (Acetylsalicylic acid)
157		Salicil Vet® (Acetylsalicylic acid)
158		Finadyne® (Flunixin meglumina), Salicil Vet® (Acetylsalicylic acid)
159		Salicil Vet® (Acetylsalicylic acid)
160		Salicil Vet® (Acetylsalicylic acid)
161	Metacam® (Meloxicam)	Salicil Vet® (Acetylsalicylic acid)
162		Salicil Vet® (Acetylsalicylic acid)
163	Metacam® (Meloxicam)	Salicil Vet® (Acetylsalicylic acid)
164	Metacam® (Meloxicam)	Salicil Vet® (Acetylsalicylic acid)
165	Metacam® (Meloxicam)	Salicil Vet® (Acetylsalicylic acid)
166	Salicil Vet® (Acetylsalicylic acid)	
167	Metacam® (Meloxicam)	Salicil Vet® (Acetylsalicylic acid)
168		Salicil Vet® (Acetylsalicylic acid)
169		Salicil Vet® (Acetylsalicylic acid)
170		Salicil Vet® (Acetylsalicylic acid)
171	Salicil Vet® (Acetylsalicylic acid)	
172	Salicil Vet® (Acetylsalicylic acid)	
173	Salicil Vet® (Acetylsalicylic acid)	
174	Salicil Vet® (Acetylsalicylic acid)	
175	Salicil Vet® (Acetylsalicylic acid)	
176	Metacam®(Meloxicam) Salicil Vet® (Acetylsalicylic acid)	
177	Salicil Vet® (Acetylsalicylic acid)	
178	Metacam® (Meloxicam) Salicil Vet®	
179	Salicil Vet® (Acetylsalicylic acid)	
180	Salicil Vet® (Acetylsalicylic acid)	
181	Salicil Vet® (Acetylsalicylic acid)	
182	Salicil Vet® (Acetylsalicylic acid)	
183	Salicil Vet® (Acetylsalicylic acid) Finadyne® (Flunixin meglumina)	
184	Salicil Vet® (Acetylsalicylic acid)	
185	Salicil Vet® (Acetylsalicylic acid)	Dinalgen® (Ketoprofen), Finadyne® (Flunixin meglumina)
186	Metacam® (Meloxicam), Salicil Vet® (Acetylsalicylic acid)t	Dinalgen® (Ketoprofen)
187	Salicil Vet® (Acetylsalicylic acid)	
188	Salicil Vet® (Acetylsalicylic acid)	
189	Salicil Vet® (Acetylsalicylic acid)	Dinalgen® (Ketoprofen)
190	Metacam®(Meloxicam), Salicil Vet® (Acetylsalicylic acid)	Dinalgen® (Ketoprofen)
191	Salicil Vet® (Acetylsalicylic acid)	
192	Salicil Vet® (Acetylsalicylic acid)	

193	Metacam® (Meloxicam)	Salicil Vet® (Acetylsalicylic acid)
194	Finadyne® (Flunixin meglumina)	Dinalgen® (Ketoprofen), Finadyne® (Flunixin meglumina), Metacam®(Meloxicam)
195	Finadyne® (Flunixin meglumina)	Dinalgen® (Ketoprofen)
196		Salicil Vet® (Acetylsalicylic acid)
197	Metacam® (Meloxicam)	Finadyne® (Flunixin meglumina)
198		Salicil Vet® (Acetylsalicylic acid)
199		Dinalgen® (Ketoprofen), Finadyne® (Flunixin meglumina), Salicil Vet® (Acetylsalicylic acid)
200	Metacam® (Meloxicam)	Dinalgen® (Ketoprofen), Salicil Vet® (Acetylsalicylic acid)
201	Metacam® (Meloxicam)	Salicil Vet® (Acetylsalicylic acid)
202		
203		
204	Finadyne® (Flunixin meglumina)	
205	Metacam® (Meloxicam)	
206		Finadyne® (Flunixin meglumina)
207	Metacam® (Meloxicam)	
208		Finadyne® (Flunixin meglumina)
209		Salicil Vet® (Acetylsalicylic acid)
210		Salicil Vet® (Acetylsalicylic acid)
211		Salicil Vet® (Acetylsalicylic acid)
212	Finadyne® (Flunixin meglumina), Metacam®(Meloxicam)	Salicil Vet® (Acetylsalicylic acid)
213	Metacam® (Meloxicam)	Salicil Vet® (Acetylsalicylic acid)
214	Metacam® (Meloxicam)	Finadyne® (Flunixin meglumina), Salicil Vet® (Acetylsalicylic acid)
215	Metacam® (Meloxicam)	Finadyne® (Flunixin meglumina), Salicil Vet® (Acetylsalicylic acid)
216		Finadyne® (Flunixin meglumina), Salicil Vet® (Acetylsalicylic acid)
217	Finadyne® (Flunixin meglumina),Salicil Vet® (Acetylsalicylic acid)	
218	Salicil Vet® (Acetylsalicylic acid)	Finadyne® (Flunixin meglumina)
219		
220	Metacam®(Meloxicam) Salicil Vet® (Acetylsalicylic acid)	
221	Metacam® (Meloxicam), Salicil Vet® (Acetylsalicylic acid)	
222	Metacam®(Meloxicam), Salicil Vet® (Acetylsalicylic acid)	Finadyne® (Flunixin meglumina)
223	Metacam®(Meloxicam), Salicil Vet® (Acetylsalicylic acid)	Finadyne® (Flunixin meglumina)
224	Salicil Vet® (Acetylsalicylic acid)	Finadyne® (Flunixin meglumina)
225	Salicil Vet® (Acetylsalicylic acid)	
226	Salicil Vet® (Acetylsalicylic acid)	

227	Salicil Vet® (Acetylsalicylic acid)	
228	Salicil Vet® (Acetylsalicylic acid), Metacam®(Meloxicam)	Finadyne® (Flunixin meglumina)
229	Salicil Vet® (Acetylsalicylic acid)	
230		
231		
232		
233		
234		
235		
236		
237	Metacam® (Meloxicam)	
238		
239	Metacam® (Meloxicam)	Finadyne® (Flunixin meglumina)
240	Metacam® (Meloxicam)	
242	Metacam® (Meloxicam)	Finadyne® (Flunixin meglumina)
242	Metacam® (Meloxicam)	Finadyne® (Flunixin meglumina)
243	Finadyne® (Flunixin meglumina), Metacam® (Meloxicam)	Finadyne® (Flunixin meglumina)
244	Finadyne®, Metacam®(Meloxicam)	
245	Finadyne® (Flunixin meglumina)	Salicil Vet® (Acetylsalicylic acid)
246	Finadyne® (Flunixin meglumina)	Salicil Vet® (Acetylsalicylic acid)
247	Finadyne® (Flunixin meglumina)	
248	Finadyne® (Flunixin meglumina)	Salicil Vet® (Acetylsalicylic acid)
249		Salicil Vet® (Acetylsalicylic acid)
250	Finadyne® (Flunixin meglumina)	Finadyne® (Flunixin meglumina), Salicil Vet® (Acetylsalicylic acid)
251	Metacam®(Meloxicam)	Salicil Vet® (Acetylsalicylic acid)
252	Metacam® (Meloxicam)	Finadyne® (Flunixin meglumina), Salicil Vet® (Acetylsalicylic acid)
253		Salicil Vet® (Acetylsalicylic acid)
254		Finadyne® (Flunixin meglumina), Salicil Vet® (Acetylsalicylic acid)
255	Metacam® (Meloxicam)	Finadyne® (Flunixin meglumina),Salicil Vet® (Acetylsalicylic acid)
256	Metacam® (Meloxicam)	Finadyne® (Flunixin meglumina) Salicil Vet® (Acetylsalicylic acid)
257		Salicil Vet® (Acetylsalicylic acid)
258		Salicil Vet® (Acetylsalicylic acid)
259	Metacam® (Meloxicam)	Salicil Vet® (Acetylsalicylic acid)
260		Salicil Vet® (Acetylsalicylic acid)
261		Salicil Vet® (Acetylsalicylic acid)
262		Salicil Vet® (Acetylsalicylic acid)
263		Finadyne® (Flunixin meglumina), Salicil Vet® (Acetylsalicylic acid)

264	Salicil Vet® (Acetylsalicylic acid)

Abbreviations: ID=animal identification, NSAID=non-steroid anti-inflammatory drug.
